# The first report of iron-rich population of adapted medicinal spinach (*Blitum virgatum* L.) compared with cultivated spinach (*Spinacia oleracea* L.)

**DOI:** 10.1038/s41598-021-01113-9

**Published:** 2021-11-12

**Authors:** Ali Ammarellou, Valiollah Mozaffarian

**Affiliations:** 1 Research Institute of Modern Biological Techniques, University of Zanjan, Zanjan, Iran; 2 Research Institute of Forests and Rangelands, Tehran, Iran

**Keywords:** Biological techniques, Ecology, Physiology, Plant sciences

## Abstract

Folk medicine such as herbal and natural products have been used for centuries in every culture throughout the world. The Chenopodiaceae family with more than 1500 species is dispersed worldwide. The Iranian wild spinach (*Blitum virgatum* L.) is an important traditional medicinal plant used for antiviral diseases such as pneumonia and other respiratory track infections. This plant is a mountainous herb and is growing upper than 3000 m. We performed a mass selection plant breeding program on wild populations of this Iranian wild spinach during 2013–2020. Based on experimental and field characteristics this plant was identified as *B. virgatum*, |*abbaricum|,* and related characteristics were prepared with reference to the International Union for the Protection of New Varieties of Plants (UPOV). Mass selection program resulted from an adapted population named as medicinal spinach (MSP) population. To compare the mineral content of the mass-selected population with cultivated spinach (*Spinacia oleracea* L*.* |Varamin 88|), both plants were planted in pots and fields under similar conditions. In five leaves stage, plant samples were taken from both leaf and crown sections and used for experimental analysis. Atomic absorption spectroscopy was used to determine the mineral content including iron (Fe), zinc (Z), manganese (Mn), and copper (Cu). Our results showed the selected medicinal spinach population (MSP) with about 509 ppm iron was an important iron-rich population with about 3.5–4 times more than the amount of iron in cultivated spinach in the same conditions. Because iron is an important essential element for blood production, respiration process, energy metabolisms, synthesis of collagen, and some neurotransmitters are needed for proper immune function, so the supply of absorbable adequate iron is very important. The reasons such as the prevalence of the COVID-19 pandemic, which affects the amount of exchangeable oxygen in the lungs and historical local evidences of the use of this plant (MSP) for pneumonia, could open new horizons for focusing on studies related to the use of ancestral human experiences in addition to scientifically modern research.

## Introduction

Plant breeding ideas and programs, which started with the domestication of wild and weedy plant species about 10,000 years ago, have played an important role in providing and supplying the food, feed, fuel, and fiber needs for the development of human civilizations to sustaining more than 6 billion humans, that living in the world now^1^. Plant breeding has been defined and explained by various researchers over the last hundred years^2^. Some of the concepts of plant breeding in the minds of these numerous breeders can be categorized as follows: the breeding of plants is an exercise in exploiting the genetic system^3^; application of techniques for exploiting the genetic potential of plants^4^; art and science of improving humankind^5^; science, art, and business of improving plants for human benefit^6^; and art and science of improving the genetic pattern of plants related to their economic uses^1^. The longest and continuous plant section method is phenotypically selection. It is a simple method that requires minimum resources and has been effective in many instances. The greatest contributions and practical results from phenotypic selection have to include making the transition from the wild, weedy plant species to cultivated crop species^1^. The primarily plant breeders had more effective role in the development of productive cultivated plant species from wild species that were lower in productivity but had many important traits such as drought tolerance and pest resistance which were necessary for their survival in the wild habitats^1,2,6^.

The mass selection program as the oldest and important method in plant breeding applied by a primitive humans during the hundreds of generation of plants in the world^1,7^. The development of new domesticated and cultivated species from wild ancestors is due to the function of mass selection. The success and efficiency of this method depend on several factors, including phenotype and performance. The resulted seeds from selected plants are bulked for the next generation. This method is used to overall improve the plant populations by positive or negative selections. Mass selection is an effective method for the improvement of land races^1^. This method of selection is commonly based on genetics and will only be effective for highly heritable traits^1,8^. One of the limitations of mass selection is the large influence of the environment on the growth, phenotype, and yield of individual plants. It can also be an advantage that different types can be selected for local performance^8,9^.

The Chenopodiaceae family with more than 1500 species, including vegetable crops such as spinach, beets, and desert plants like Atriplex (saltbush) is dispersed worldwide. Many chenopod species have C4 photosynthesis^10,11^.

Iran is the early center of spinach diversity and its wild species grow as a wild vegetables in many of Iran’s mountains, including the northern slopes of the Alborz and Zagros, whose identification can lead to the introduction of high-yielding populations and disease-resistant ones^12,13^. The Iranian wild spinaches are the important traditional medicinal plants used for antiviral diseases such as pneumonia and other respiratory track infections.

Iron deficiency in humans may cause anemia, which can probably reduce activity and resistance to fatigue. In pregnant women, iron-deficiency anemia is associated with an increased risk of maternal mortality, fetal growth retardation, premature births, low birth weight and high susceptibility to infections, and even mortality^14^.

This study aims to assess the mineral contents of two spinach including mass-selected Medicinal Spinach (MSP) and common Cultivated Spinach (CSP). Popular belief in the world recognizes cultivated spinach as one of the iron-rich vegetables. In this study, we introduced another mass-selected population of spinach that is richer Iron than it.

## Materials and methods

### Collection and domestication of the wild populations

The academic permission for collections and research on medicinal plants was obtained from the Head of Biotechnology Department, Research Institute of Modern Biological Techniques, University of Zanjan, Zanjan, Iran. The study complies with all relevant guidelines. Some populations of wild spinaches were harvested during spring season 2013 from the mountain habitat of this wild plant in the Tarom region of Zanjan province from an altitude of 2500–3000 m and were transferred to the greenhouses conditions. The domestication and cultivation experiments were conducted at Research Institute of Modern Biological Techniques, University of Zanjan, 1579° m above sea level, with 48° 28′ longitude and 36° 40′ latitude, from April 2013 to August 2020. The resulted seeds were cultured on pots to produce adequate seeds. The seedlings were transferred to the field with rows spaced 50 cm apart and also 50 cm between plants within the rows. Two seeds per hill were planted in an area of approximately 50 m^2^. Based on the organic conditions, no fertilization was performed. Thinning was done 25 days after emergence, leaving one plant per hill. The other cultural practices were those normally adopted for cultivation in the region.

### Mass selection of populations

In the first year, phenotypic studies were performed during the growing season and weak, diseased and underdeveloped plants were removed from the field before the flowering stage. Then plants with the same phenotype and the desired traits were selected and after harvesting, their seeds were mixed. This election cycle was repeated for 5 years. In the final year, the new mass selected population was compared in a pilot project with cultivated spinach in traits such as yield, resistance to wilt, cold and pests, diseases, and mineral contents. This variety before the certification in the related national organization is a candida cultivar. It is a developed population that will be evaluated in the session of the Iranian variety of introduction committee.

The seeds of cultivated spinach (*Spinacia oleracea* L*.* |Varamin 88|) were prepared from the Research Institute of Modern Biological Techniques, University of Zanjan, Zanjan, Iran.

### Performing tests of stability, uniformity and differentiation

To assess morphologically and differentiate advanced uniformity in the studied population (Candida cultivar), the population was managed as a randomized complete block design with three replications over 2 years according to the instructions for spinach differentiation, uniformity, and stability (DUS Testing) of the International Union New Plant Cultivation (UPOV) and some morphological traits on plants or parts of plants. The studied traits included: cotyledon length, presence or absence of anthocyanin in petiole and veins, green color intensity, shrinkage, presence of lobes in the petiole, petiole state, petiole length, foil shape, foil edge shape, tip shape, and part of the length of the petiole, the time of flowering and the color of the seeds.

### Mineral analyses

To compare the mineral content of mass-selected population-medicinal spinach (MSP) with cultivated spinach (*Spinacia oleracea* L. var. Varamin 88), both plants were planted in pots and fields on similar conditions. In five leaves stage, plant samples were taken from both leaf and crown sections. The sampling method was such that after removing half a meter from the beginning and end of each plot (to remove the marginal effect) and also removing the two sidelines, five plants were harvested randomly for plant mineral analysis. Atomic absorption spectroscopy was used to determine the mineral content including iron (Fe), zinc (Z), manganese (Mn), and copper (Cu).

The dried samples of root-crown and leave were stored, and later grounded and analyzed for iron (Fe), zinc (Z), manganese (Mn), and copper (Cu) in mass-selected variety (MSP) and cultivated spinach (CSP). Studied minerals were measured using atomic absorption spectrometry in the model of GBC AVANTA *(*GBC scientific equipment Ltd., Melbourne, Vic., Australia).

Calibration of AAS was done using the working standard prepared from commercially available metal/mineral standard solutions (1000 μg/mL, Merck, Germany). The most appropriate wavelength, hollow cathode lamp current, gas mixture flow rate, slit width, and other AAS instrument parameters for metals/minerals were selected as given in the instrument user’s manual, and background correction was used during the determination of metals/minerals. Measurements were made within the linear range of working standards used for calibration^15,16^.

The concentrations of all the minerals were expressed as mg/1000 g (ppm) dry weight of the sample. Each value is the mean of three replicate determination ± standard deviation.

### Scanning electron microscopy (SEM)

For SEM studies, the seeds enveloping were removed and were acetolyzed in a 1:9 sulfuric acid-acetic anhydride solution. The seeds were vigorously shaken for 5 min. Then, they were left for 24–48 h in the solution. After this time, seeds were again shaken for 5 min and then washed.

in distilled water by shaking for a further 5 min. The seeds were dried overnight and then were mounted on stubs and covered with Au–Pd by sputter coater model SC 7620. After coating, coated seeds were photographed with an LEO 1450 VP Scanning Electron Microscope. All photographs were taken in the Taban laboratory (Tehran, Iran).

### Statistical analysis

The statistical evaluation including: data transformation, analysis of variance and comparison of means were performed (SPSS software, Version 11.0). The experiment was structured following a randomized complete block design (RCBD) with three replications. Means comparisons were conducted using an ANOVA protected the least significant difference (LSD) test, with the ANOVA confidence levels of 0.95. Data were presented with their standard deviations (SD).

## Results and discussion

**Figure 1:**
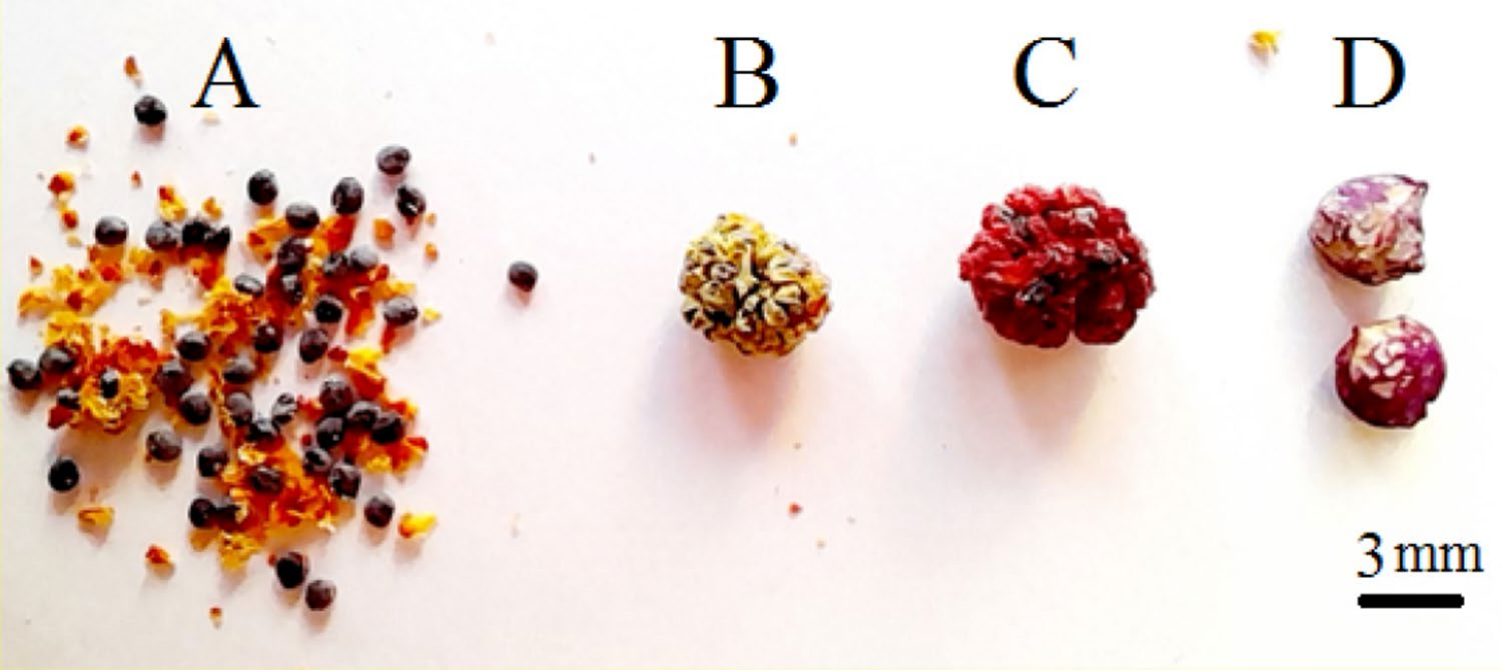
The comparison of seed size, seed color between *B. virgatum* [(**A**) dried seed, (**B**) immature dried fruits, (**C**) matured dried fruit] and commonly cultivated spinach seeds (**D**).

**Figure 2:**
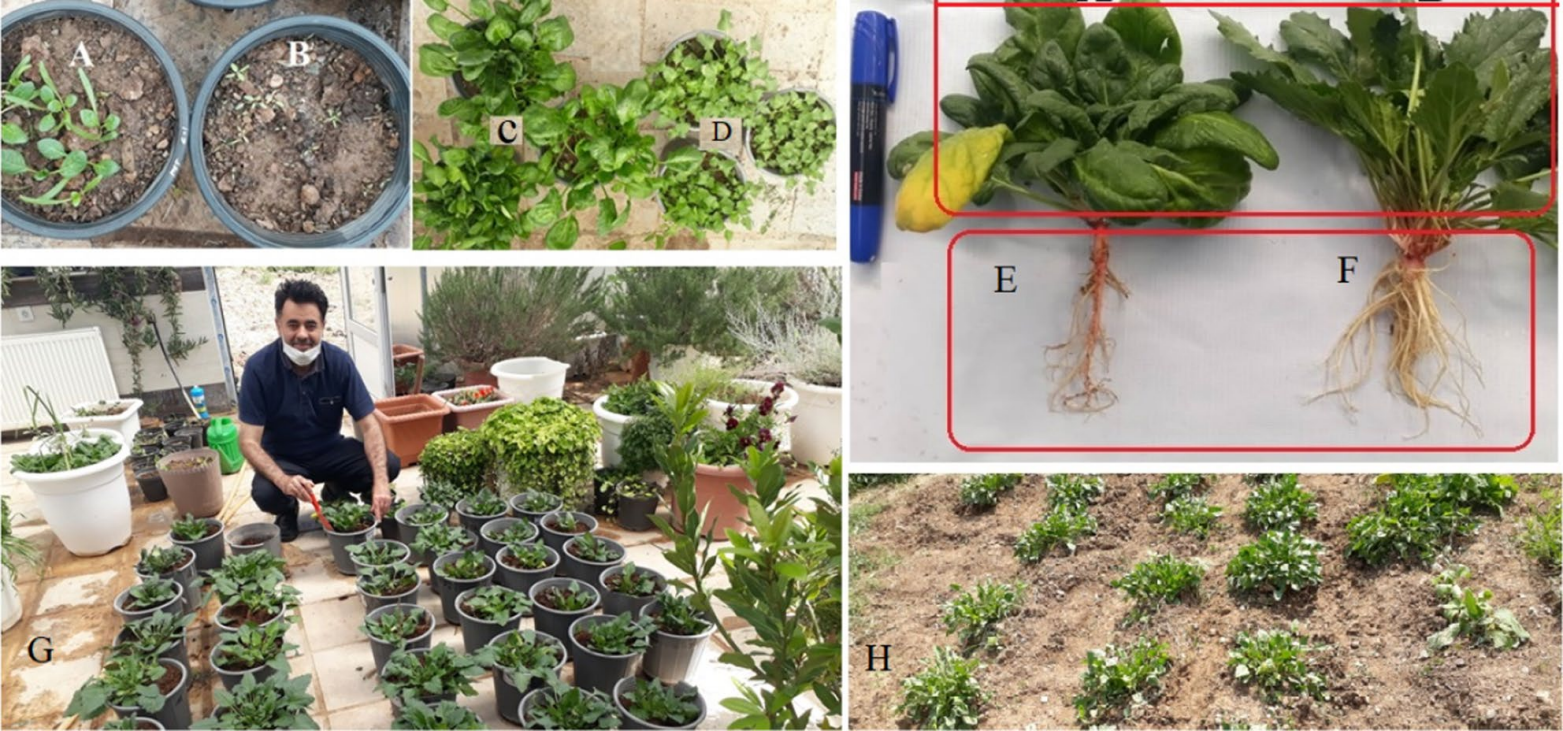
Seedlings of two studied plants [(**A**) cultivated spinach, (**B**) wild spinach] and greenhouse experiments [(**C**) cultivated spinach, (**D**) wild spinach]. Morphological characteristics of complete plants [(**E**) cultivated spinach and (**F**) domesticated spinach]. Some agronomical practices on domesticated spinach (**G**), and field culture of selected spinach for the start of mass selection program (**H**). The person in G is Ali Ammarellou, author and researcher of this project.

**Figure 3:**
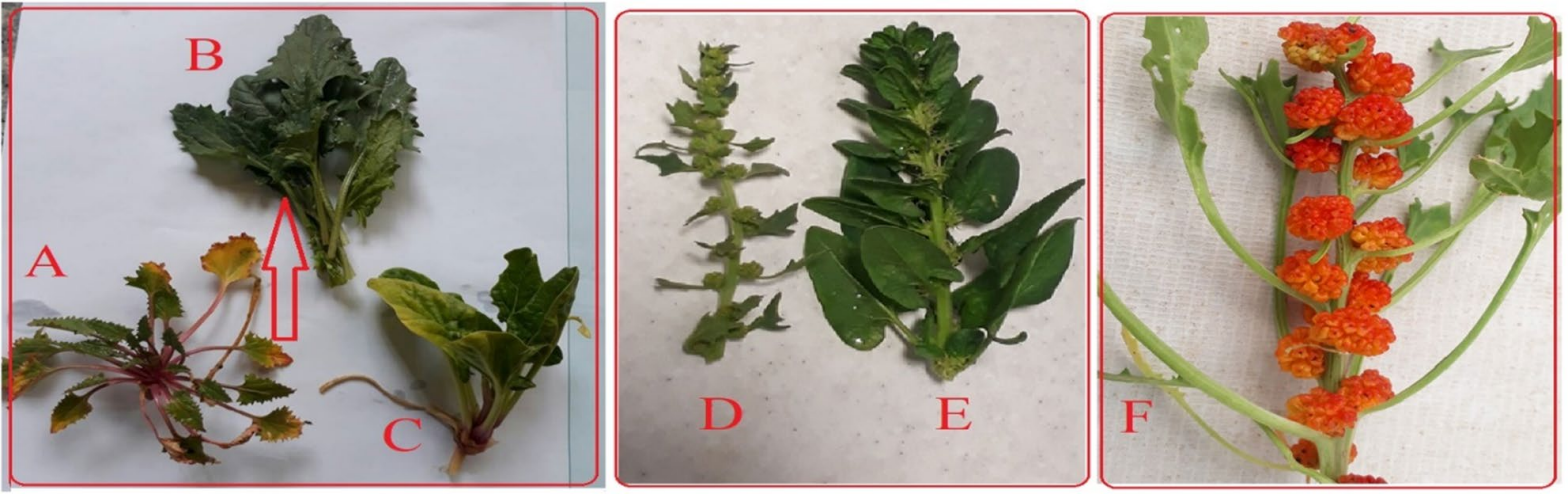
The sample of mother wild population (**A**) and mass-selected domesticated population (**B**), the common spinach-Varamin 88 (**C**), flowering stem in domesticated spinach (**D**), flowering stem in cultivated spinach (**E**), and mature fruits of mass selected spinach (**F**).

**Figure 4:**
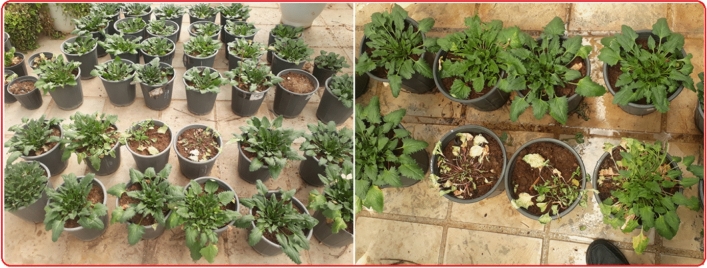
Some greenhouse studies for adaptation of wild spinach populations to put culture conditions. Some plants and genotypes are not able to adapt to agronomic conditions that dry out and wither after a while. About 20 percent of the study population in every generation could not adapt to non-mountainous and non-habitat conditions.

**Figure 5:**
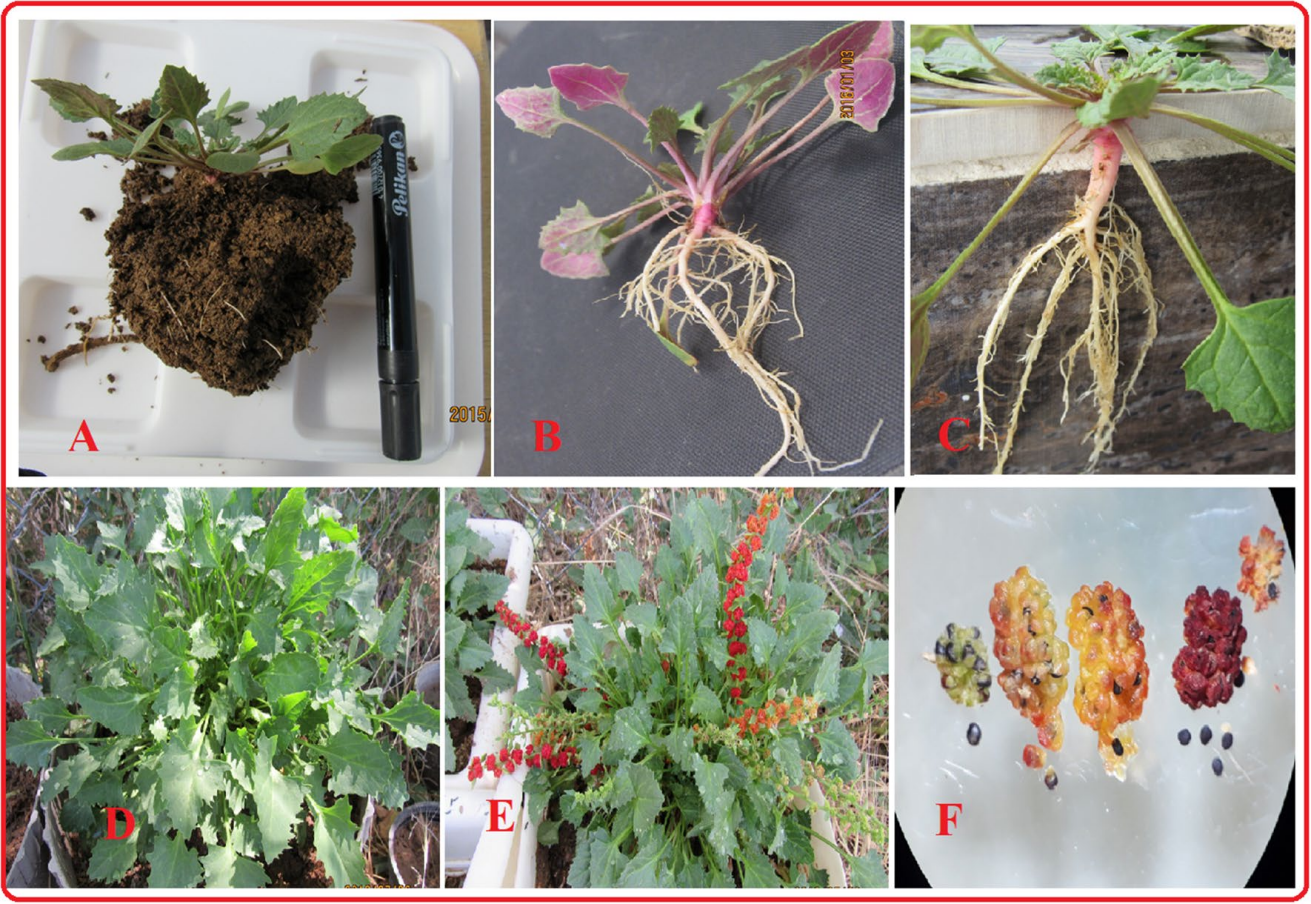
Different stages of greenhouse growing of mass-selected variety. (**A**) Soil profile of seedling, (**B**, **C**) complete seedling of MSP, (**D**) late of the growing stage, (**E**) fruiting stage, (**F**) development of seeds in fruits.

**Figure 6:**
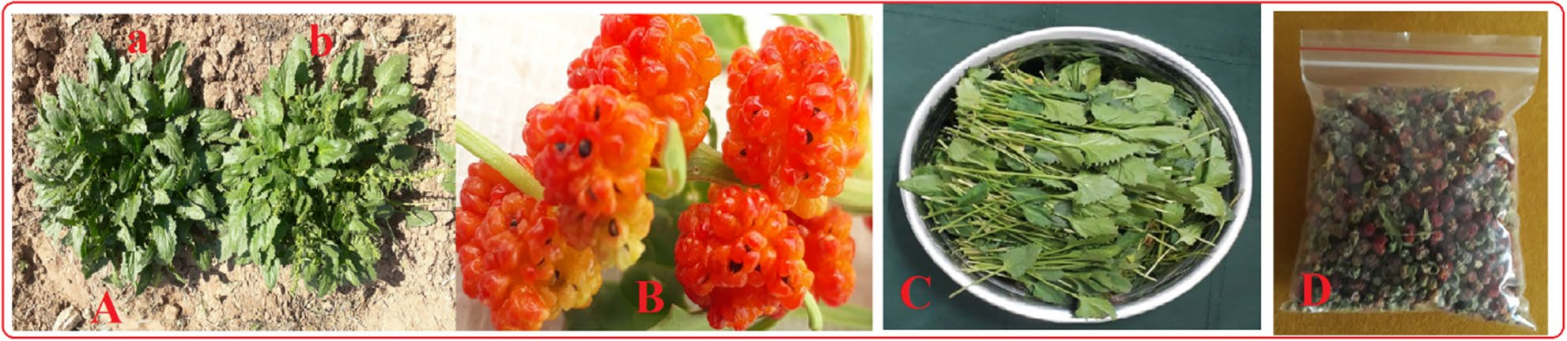
The high yield variety (MSP) resulted from 7 years field mass selection program [(**A**) a: vegetative stage and b: generative stage], matured fruits (**B**), harvested leaves of MSP (**C**), and seeds of introduced variety (**D**).

**Figure 7:**
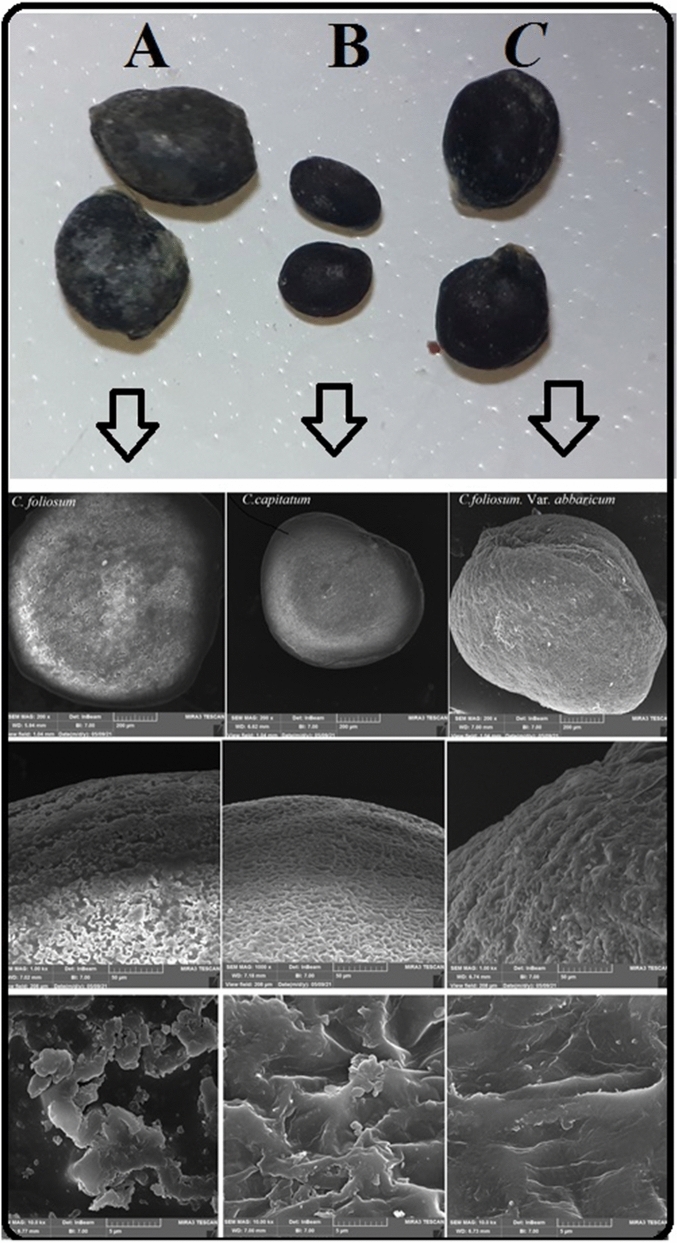
Differences in seed coat ornamentals in the three species studied by scanning electron microscopy (SEM). (**A**) *Chenopodium foliosum* (*syn. Blitum virgatum*), (**B**) *Chenopodium capitatum* & (**C**) *Chenopodium foliosum |abbaricum|* (MSP). The SEM characteristics were used for identifying three plants.

The comparison of seed characteristics between *B. virgatum,|abbaricum*| and commonly cultivated spinach were presented in Fig. 1. Figure 2 shows part of the germination experiments and greenhouse studies of the two studied species. The seeds of cultivated spinach (*Spinacia oleracea* L*.*) are larger than wild spinach and produce stronger plants. Based on morphological characteristics and growth cycle studies, the studied wild medicinal spinach was identified as *Blitum virgatum* L. |*abbaricum|*^17^. This species is a hermaphrodite plant (which has both male and female organs of flower) and is pollinated by wind. It is an erect annual herb growing to a maximum height of just over half a meter. The leaves are 1–4 cm long (depend on growth stages) and maybe toothed or smooth-edged. The inflorescences are small spherical clusters of tiny reddish-green flowers wrapped around fruits which are about a millimeter wide (Figs. 2, 3, 4, 5, 6 and 7). The comparative data of the two studied genera were presented in Tables 1 and 2.

**Table 1:** Appearance differences and morphological characteristics of cultivated spinach (CSP) and medicinal spinach (MSP).

Plant	Seed color	Seed size (mm)	Germination (day)	Leaf shape	Leaf color	Crown diameter (cm)	Root system
Cultivated spinach (CSP)	Light red	2–3	7–10	Round to elongated	Light green	1–2	Tap root
Medicinal spinach (MSP)	Brown to black	0.2–0.7	25–35	Sharp and thin	Dark green	3–5	Strong tap root

**Table 2:** Identification of medicinal spinach (MSP) according to the instructions of DUS testing spinach distinctness, uniformity, and stability-reference to UPOV^18^.

Number	Characteristics	Appearance	Code
1	Seedling: length of cotyledon	Small	3
2	Leaf (anthocyanin)	Exist	5
3	Leaf color	Dark green	5
4	Leaf blisters	No exist	1
5	Leaf: the presence of lobes	No exist	1
6	Petiole: mode	Semi-erect	3
7	Petiole: long	Medium	5
8	Leaf blade: mode	Semi-erect	3
9	Leaf blade: shape	Triangular or spear	6
10	Leaf blade: shape of apex	Acute	1
11	Leaf blade: leaf edge curvature	Rounded	2
12	Leaf-blade: the shape of the longitudinal part of the leaf	Smooth	2
13	Proportion of monoecious plants	Very high	9
14	Proportion of female plants	Absent or very low	1
15	Proportion of male plants	Absent or very low	1
16	Time of start of bolting (for spring sown crops, 15% of plants)	Medium	5
17	Seed: spines (harvested seed)	Absent	1

The resulted seeds from seven cycles of mass selection (Fig. 6D) were mixed and introduced as MSP variety. The Voucher specimens of this variety were registered and stored in the Plant Herbarium of Research Institute of Modern Biological Techniques-University of Zanjan, Iran, with code access number: RIMBT-MP-1400. Our results showed the selected population (MSP) with about 509 ppm iron in crown-root was an important iron-rich population with about 3.5–4 times more than the amount of iron in cultivated spinach (CSP) (Table 3).

**Table 3:** Concentration (ppm or mg/kg-dry matter) of some major minerals including iron (Fe), zinc (Z), manganese (Mn), copper (Cu) in mass-selected variety (MSP) and cultivated spinach (CSP).

Elements^a^	MSP-root	MSP-leaf	CSP-root	CSP-leaf
Iron	508.9 ± 8	107.9 ± 3	144.4 ± 4	66.8 ± 2
Manganese	32.6 ± 1	22.6 ± 2	22.1 ± 1	33.2 ± 1
Zinc	56.8 ± 3	50 ± 2	54.1 ± 2	79.5 ± 2
Copper	8.9 ± 0.1	8.4 ± 0.2	7.4 ± 0.3	6.3 ± 0.1

^a^Each value is the mean of three replicate determinations ± standard deviation.

Medicinal plants are a more important source of secondary metabolites and essential oils of therapeutic importance. The important advantages claimed for therapeutic uses of medicinal plants in various ailments are their safety besides being economical, effective, and easy availability^19^. Spinach originates from south-eastern Asia, and Ancient Persia, where first cultivated about 2000 ago. Nowadays it is one of the most widespread and consumed leafy green vegetables all over the world. Spinach is a mineral-rich vegetable. Some reports showed the average amount of iron in this plant is about 10–11 mg/100 g dry matter^20^. General believe is think about spinach as a rich-iron plant. Although the available information challenges this belief, the introduction of a variety of its family with about 3.5–4 times iron more than cultivated spinach has fulfilled this old dream.

Because Iron is an important essential element for blood production, respiration process, energy metabolisms, synthesis of collagen and some neurotransmitters, and is needed for proper immune function^14^, so the supply of absorbable adequate iron is very important. The reasons such as the prevalence of the COVID-19 pandemic, which affects the amount of exchangeable oxygen in the lungs and historical local evidence of the use of this plant (MSP) for pneumonia, could open new horizons for focusing on studies related to the use of ancestral human experiences beside the scientific modern research.
